# Activation of the Mammalian Target of Rapamycin Pathway in Endothelial Cells in Antiphospholipid Antibody-Positive Patients with Leg Ulcers

**DOI:** 10.3390/ijms26062750

**Published:** 2025-03-19

**Authors:** András L. Kovács, Csaba Gyömörei, Szabina Horváth, Viktória Németh, Réka Dudley, Zsuzsanna Nagy, Tímea Berki, Zsuzsanna Lengyel, Rolland Gyulai

**Affiliations:** 1 Department of Dermatology, Venereology and Oncodermatology, Medical School, University of Pécs, 7622 Pécs, Hungary; kovacs.l.andras@pte.hu (A.L.K.); horvath.szabina@pte.hu (S.H.); nemeth.viktoria@pte.hu (V.N.); lengyel.zsuzsanna@pte.hu (Z.L.); 2 Department of Pathology, Medical School, University of Pécs, 7622 Pécs, Hungary; gyomorei.csaba@pte.hu; 3 Department of Dermatology, Veszprém County Csolnoky Ferenc Hospital, 8200 Veszprém, Hungary; dudleyreka2@gmail.com; 4 2nd Department of Internal Medicine and Nephrological Center, Medical School, University of Pécs, 7622 Pécs, Hungary; zsuzsanna.nagy69@gmail.com; 5 Department of Immunology and Biotechnology, Medical School, University of Pécs, 7622 Pécs, Hungary; berki.timea@pte.hu; 6 Department of Dermatology and Allergology, Albert-Szent Györgyi Medical School, University of Szeged, 6720 Szeged, Hungary

**Keywords:** antiphospholipid syndrome, antiphospholipid antibody, mammalian target of rapamycin, leg ulcer

## Abstract

Antiphospholipid antibody (aPL)-induced activation of the mTOR (mammalian target of rapamycin) signaling pathway in endothelial cells plays a role in the pathogenesis of vascular lesions in antiphospholipid syndrome (APS). However, there are no data on whether this mechanism also contributes to the development of skin ulcers commonly observed in APS. We investigated the activation of mTOR in skin specimens from aPL-positive and aPL-negative patients with leg ulcers. Patients with leg ulcers who had primary or secondary APS or no detectable aPLs were included in the study. Biopsies were taken from the ulcer edges and the adjacent non-ulcerated skin areas. Activation of mTORC1 (mTOR Complex1) and mTORC2 (mTOR Complex2) in endothelial cells was determined by immunohistochemical analysis of phosphorylated ribosomal S6 protein (pS6RP) and phosphorylated protein kinase B (pAKT), respectively. In all aPL-positive patients, regardless of whether they had primary or secondary APS, we found a positive immunohistochemical reaction to pS6RP (mTORC1 activation) in the endothelial cells of the ulcer samples. On the other hand, pS6RP could not be detected in samples from aPL-negative chronic venous ulcers. Furthermore, pS6RP was not present in samples taken from the unaffected skin adjacent to the ulcers in aPL-positive patients. The pAKT reaction (mTORC2) was negative in both aPL-positive and aPL-negative patients, both in the ulcers and in the periulcer skin. Activation of the mTOR pathway may contribute to ulcer development in APS. The mTORC1 may be a target for therapeutic modification in APS-associated skin ulcers.

## 1. Introduction

Antiphospholipid syndrome (APS) is a systemic autoimmune disease characterized by persistently elevated levels of circulating antiphospholipid antibodies (aPL), such as lupus anticoagulant (LAC), anticardiolipin antibodies (aCL), and anti–β2 glycoprotein I (aβ2GPI) antibodies. Clinically, the disease is characterized by the occurrence of venous, arterial, and microvascular thrombosis, along with spontaneous miscarriages and other pregnancy-related morbidities [1,2]. APS can present as primary APS without associated comorbidities, but it can also occur alongside a broad spectrum of autoimmune diseases (secondary APS) [3]. A diagnosis of APS requires a history of one or more thrombotic events or obstetric complications, and the fulfillment of at least one clinical and one laboratory classification criterion from the revised Sapporo criteria [4]. The binding of aPLs to β2GPI results in the activation of platelets, neutrophil granulocytes, monocytes, the complement system, and endothelial cells, thereby shifting the intravascular equilibrium toward in situ thrombosis [4,5]. In addition, in APS, severe non-thrombotic microvascular complications (occlusive vasculopathy) can also develop in the kidneys, heart, lungs, and skin, potentially leading to progressive organ damage over time, such as graft loss following kidney transplantation [2,4,6,7,8,9].

A wide range of skin manifestations, occurring in approximately half of patients, is associated with APS. In about one-third of cases, these may even present as the first sign of the syndrome [6]. Microcirculatory disturbances, post-thrombotic syndrome, and a decrease in tissue oxygen pressure may lead to the development of leg ulcers in APS [10,11]. Recent findings suggest that the mTOR signaling pathway may be involved in the vascular lesions associated with antiphospholipid syndrome, as aPLs bind to renal vascular endothelial cells and activate the mTOR complex [12,13]. In addition, increased mTOR activity has been observed in samples from both central and peripheral regions of livedo reticularis skin lesions in aPL-positive systemic lupus erythematosus (SLE) and aPL-positive non-SLE patients [14]. mTOR protein kinase forms two large, structurally and functionally distinct multiprotein complexes in cells: mTORC1 and mTORC2. These complexes are not only structurally distinct but also differ in their regulation, their targets, and the cellular processes they control. The target proteins of mTORC1 are ribosomal S6 kinase (S6K) and 4E-binding protein (4EBP). The target of mTORC2 is AKT kinase, which undergoes phosphorylation at serine 473 (Ser 473) [15,16].

These findings suggest that the mTOR pathway may play a role in the development of lower limb ulcers in antiphospholipid syndrome; however, no research has yet been carried out into the function of mTOR in APS-associated ulcers. The present study therefore aims to investigate the activation of the mTOR pathway in APS-associated lower limb ulcers.

## 2. Results

**Figure 1:**
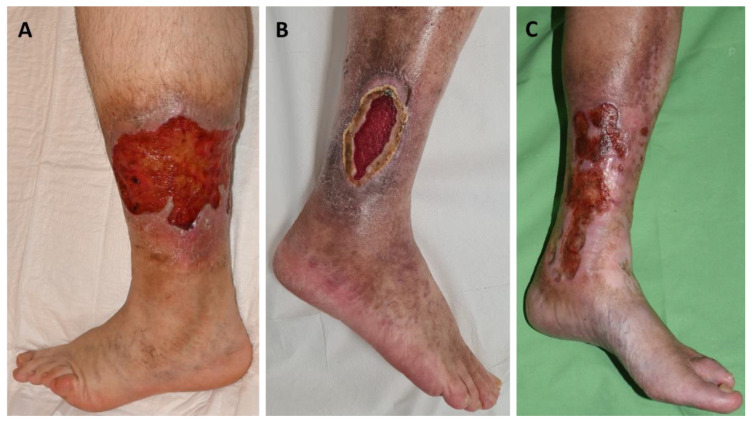
(**A**) Chronic leg ulcer in primary APS, (**B**) secondary APS, and (**C**) aPL-negative patient.

**Figure 2:**
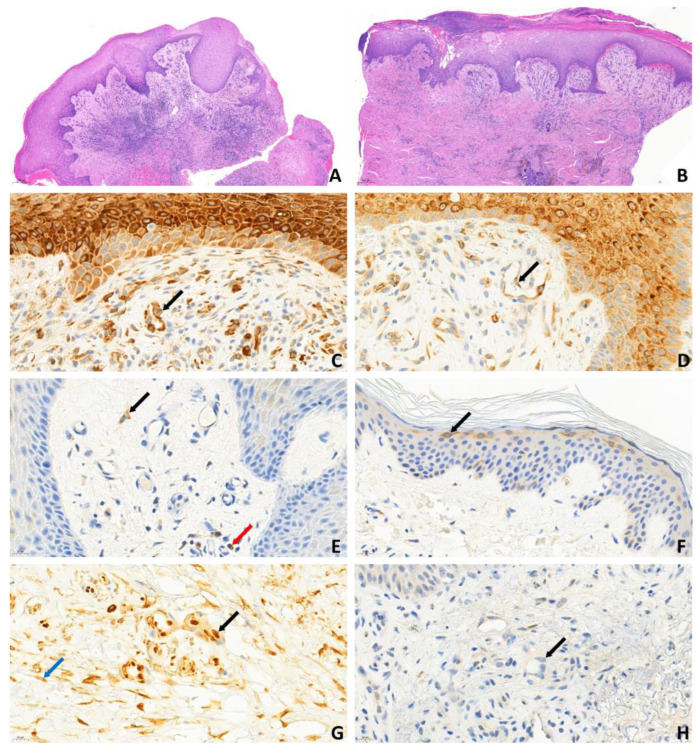
Biopsy of the ulcer margin stained with hematoxylin-eosin (5×) in (**A**) primary APS and (**B**) secondary APS. Epidermal and endothelial (black arrow) cytoplasmatic pS6RP expression in (**C**) primary APS (45×) and (**D**) secondary APS (45×). (**E**) pS6RP negativity in the ulcer of an aPL-negative patient; the brown-stained cell is a fibroblast (black arrow) and a plasma cell (red arrow) (45×). (**F**) In regions distant from the ulcer, phospho-S6 positivity (black arrow) is exclusively localized to the granular layer (45×); this figure shown the nuclear expression of the ERG endothelial marker and cytoplasmic pS6RP expression in the endothelium (black arrow). (**G**) Cells exhibiting only cytoplasmatic positivity are fibroblasts (blue arrow); ERG/pS6RP double staining (45×). (**H**) pAKT-negative endothelium (black arrow) (45×).

A total of 13 patients with leg ulcers were enrolled in this study (11 women, 2 men; all Caucasian; mean age: 60.61 years): 5 aPL-positive patients without systemic autoimmune disease, 5 aPL-positive patients with other systemic autoimmune diseases, and 3 aPL-negative patients without autoimmune disease (Table 1). All patients had chronic leg ulcers (Figure 1). Among the five patients with primary APS, all were LAC-positive, and aCL and aβ2GPI antibodies were negative. Of the five patients with secondary APS, five were LAC-positive, four aCL-positive, and four aβ2GPI-positive, and four showed triple aPL positivity. Diagnoses within this group included one case of systemic lupus erythematosus (SLE, P6), one case of mixed connective tissue disease (MCTD, P10), one patient receiving immunological care due to non-differentiated collagenosis (NDC) SLE type (P7), and two patients (P8, P9) receiving immunological care due to NDC Sjögren’s syndrome type (Table 1). Biopsies from the ulcer edges of both primary and secondary APS patients comprised hyper-parakeratosis, pseudoepitheliomatous hyperplasia, dermal fibrosis, increased vascularity, and moderate-to-dense perivascular chronic inflammatory cell infiltrate. The vessels were lined with endothelial cells with swollen nuclei, a finding consistent with vasculopathy (Figure 2A,B). Immunohistochemical analysis revealed cytoplasmic pS6RP positivity in the endothelial cells of all 10 aPL-positive patients (Figure 2C,D). There was no difference in the expression level of pS6RP between pAPS and sAPS patients. Double staining with ERG/phospho-S6 confirmed nuclear expression of the ERG endothelial marker and cytoplasmic expression of phospho-S6 within the endothelium (Figure 2G). In contrast, AKT phosphorylation was absent across all 10 aPL-positive samples (Figure 2H, Table 2). pS6RP positivity confirmed mTORC1 activity, while pAKT negativity suggested that mTORC2 was likely inactive. Interestingly, in addition to vessel positivity, S6 positivity was detected throughout the entire epithelium at the ulcer margin (Figure 2C,D). In contrast, in aPL-positive intact skin and normal (free from ulcers or other skin diseases) control samples, S6 positivity was restricted to the granular layer (Figure 2F). In samples taken from the skin adjacent to the ulcer in three aPL-positive patients, both pS6RP and pAKT were negative. Among aPL-negative controls, S6RP and AKT phosphorylation were 100% negative, indicating that the mTOR pathway was inactive in these samples (Figure 2E).

## 3. Discussion

The mTOR serine/threonine kinase protein complex plays a key role in eukaryotic cells by regulating cell growth; proliferation; survival; oxygen and energy supply; inflammatory processes; stress responses; protein, lipid, and nucleotide synthesis; and autophagy [15,17]. The regulation of mTOR activity primarily depends on metabolism and is influenced by glucose, amino acids, cytokines, growth factors, and hormones. Signals from tyrosine kinase receptors, stimulated by growth factors, are transmitted to the mTOR kinase through the PI3K (phosphatidylinositide 3-kinase) and PI3K-AKT signaling pathways [18]. Previous research has confirmed the activation of mTOR in various organ manifestations of APS. aPLs activate mTOR via the PI3K-AKT signaling pathway, significantly contributing to the development of vasculopathy observed in APS nephropathy [12]. In addition, Sevim et al. investigated mTOR activation in livedo reticularis in patients with SLE and found increased mTOR activity in skin samples from both the central and peripheral areas of livedo lesions in aPL-positive patients [14]. However, the activation of mTOR in leg ulcers associated with elevated aPL levels has not been previously studied. Here, we show that endothelial cells of small vessels in leg ulcer samples from aPL-positive patients displayed cytoplasmic S6RP positivity, while AKT phosphorylation was negative in all instances, indicating the activation of the mTOR pathway specifically through mTORC1. In the samples from aPL-negative control subjects, the activities of both the mTORC1 and mTORC2 pathways were absent. Our study confirmed increased mTOR activity in aPL-positive leg ulcers in both primary and secondary APS, suggesting that the activation of the mTOR signaling pathway may contribute to the small vessel vasculopathy observed in leg ulcers associated with APS.

Ruf et al. observed increased epidermal S6 phosphorylation in psoriasis and atopic dermatitis, suggesting general activation of the mTOR pathway in inflammatory skin diseases associated with epidermal hyperproliferation. This process may be driven by inflammatory-cell-derived cytokines (e.g., Th2 cytokines) or growth factors (e.g., insulin-like growth factor-1), which activate the mTOR pathway and inhibit keratinocyte differentiation [19]. In contrast, our present findings indicate that in non-APS-associated ulcers, despite the presence of significant inflammation and hyperproliferation, the mTOR pathway is not activated. Therefore, it is likely that inflammation and/or hyperproliferation per se are not sufficient to drive mTOR activation in leg ulcers. Alternatively, specific components (e.g., cytokines) of the inflammatory/proliferative pathways required to activate mTOR signaling could be present in APS-associated ulcers but are apparently absent in classical leg ulcers. As cytokine levels were not assessed in our study, we cannot draw any conclusions about the potential effects of the cytokine milieu on mTOR expression in APS. Furthermore, since uniformly strong mTOR expression was observed in all APS-associated ulcer samples, it is unlikely that disease severity—at least in the case of clinically established ulcers—would have an effect on the magnitude of mTOR expression.

Notably, we found that in patients with leg ulcers with elevated aPL levels, the vascular endothelium showed activation of the mTORC1 pathway, without simultaneous activation of the mTORC2 pathway. This finding differs from previous observations reported in nephropathy associated with elevated aPL levels where both mTORC1 and mTORC2 pathways were activated [12]. Sevim et al. demonstrated increased mTOR pathway activation in livedo skin epidermal cells in the absence of endothelial activation in aPL-positive patients with and without SLE, in contrast to aPL-negative patients. Their figures of peripheral and central biopsies from an aPL-positive SLE patient with livedo demonstrated heightened mTOR activity [14]. However, brown pS6RP staining showed nuclear localization, which is difficult to interpret given that cytoplasmic labeling of pS6RP would be expected.

The pAKT negativity observed in this study suggests a lack of mTORC2 activation, though the small sample size limits the significance of this observation. An investigation of its underlying cause could provide a basis for future research.

**Figure 3:**
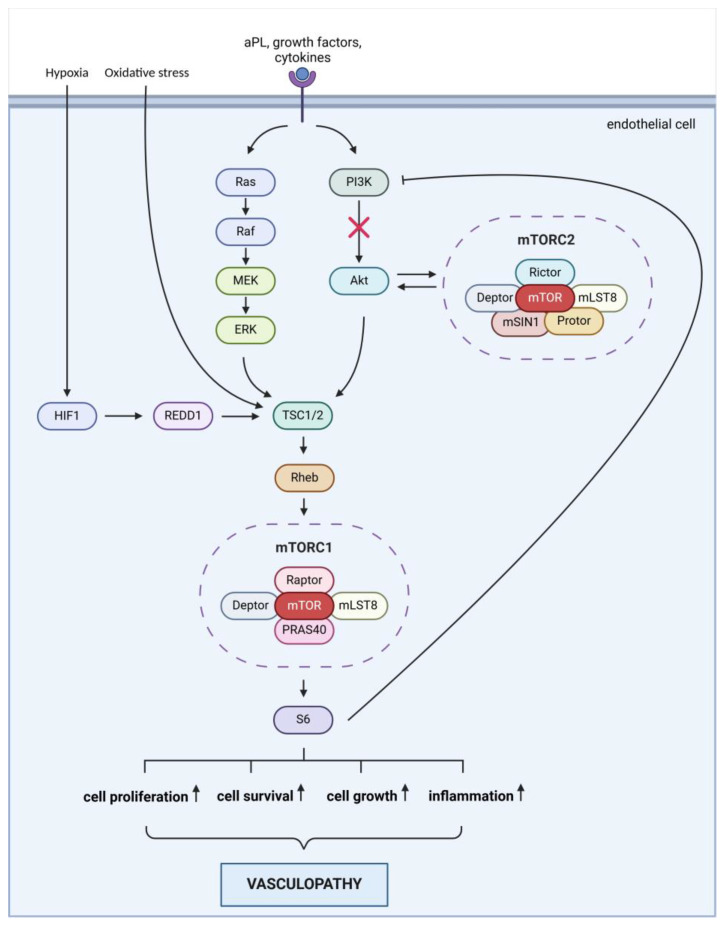
A putative schematic representation of the mTOR signaling pathway in APS. After stimulation with growth factors, cytokines, or aPL, mTORC1 can also be activated in the RAS/RAF/MEK/ERK or PI3K/AKT signaling pathways via the phosphorylation of the TSC complex and Rheb binding. pAKT negativity suggests that mTORC1 activation occurs via the RAS/RAF/MEK/ERK pathway to increase S6 protein. In addition to regulating mTORC1, pAKT is also a substrate of mTORC2; therefore, the absence of pAKT may indicate that mTORC2 is not activated. Furthermore, mTORC1 inhibits mTORC2 activation by negatively regulating PI3K/AKT signaling through the S6 protein. Increased hypoxia and oxidative stress may also enhance mTORC1 activity involving HIF-1, REDD1, the TSC complex, and Rheb protein. Active mTORC1 and elevated S6 levels mediate increased cell proliferation, cell survival, cell growth, and inflammation, which promote vasculopathy (APS, antiphospholipid syndrome; aPL, antiphospholipid antibodies; mTOR, mammalian target of rapamycin; mTORC1, mTOR complex 1; mTORC2, mTOR complex 2; Raptor, Regulatory-associated protein of mTOR; Deptor, DEP-domain-containing mTOR-interacting protein; PRAS40, Prolin-rich AKT substrate 40 kDa, mLST8, Mammalian Lethal with Sec-13 protein 8; Rictor, Rapamycin-intensive companion of mTOR; mSIN1, Mammalian stress-activated map kinase-interacting protein 1; Tel2, Telomere maintenance 2; Protor, Protein observed with rictor; Tti1, Tel2-interacting protein; PI3K, phosphatidylinositol-3-kinase; AKT, protein kinase B; MEK, mitogen-activated protein kinase; ERK, extracellular-signal-regulated kinase; TSC, tuberous sclerosis; Rheb, RAS homolog enriched in brain; REDD1, DNA damage-inducible transcript; HIF-1, hypoxia inducible factor 1; S6, ribosomal S6 protein). Created with BioRender (https://BioRender.com/h69q286 (accessed on 16 March 2025)).

The activation of the mTOR pathway in leg ulcers associated with APS may be driven by specific factors. Multiple mechanisms may contribute to the development of ulcers in APS patients (Figure 3): 1.: aPLs may directly activate the mTOR pathway by affecting the function of endothelial cells and platelets, leading to the activation of mTOR signaling. 2.: aPLs can cause endothelial dysfunction, resulting in inflammation, cell proliferation, and thrombosis. Activation of the endothelium by aPL can result in a dysfunctional state marked by reduced nitric oxide levels, while also promoting the adhesion of leukocytes and platelets. Furthermore, the activation of the complement system contributes to the proliferation of both endothelial and smooth muscle cells [5,9]. 3.: in APS, the production of inflammatory mediators such as TNF-α (tumor necrosis factor-alpha) and IL-6 (interleukin-6) is increased. These mediators can stimulate the mTOR pathway, which plays a role in perpetuating inflammation and tissue damage. 4.: APS is frequently associated with thrombosis, which leads to ischemia. The hypoxia (oxygen deficiency) that occurs due to ischemia can lead to mTOR activation through the HIF-1 (hypoxia-inducible factor 1) pathway, promoting cellular survival and adaptation. 5.: APS is also characterized by elevated oxidative stress, which can activate mTOR. Oxidative stress damages cells and exacerbates inflammation, worsening ulcer formation. Collectively, these factors may contribute to the activation of the mTOR pathway in leg ulcers associated with APS, impacting ulcer healing and tissue regeneration.

In the treatment of APS following a thromboembolic event, lifelong anticoagulation with oral vitamin K antagonists is the primary therapy. The current focus is on the prevention of thromboembolic events. Hydroxychloroquine may reduce the levels of anti-phospholipid antibodies, which play a key role in the pathogenesis of APS. However, these treatments do not prevent the vasculopathy that is involved in the development of ulcers associated with APS [9]. Rapamycin and rapalogs, by forming a complex with FKBP-12 and binding to the FRB domain of mTOR kinase, alter its conformation and inhibit mTORC1 kinase activity. In kidney transplant patients with APS, mTOR inhibitors prevent the recurrence of vasculopathy and, consequently, graft loss [12]. The inhibitory effect of sirolimus on neointimal proliferation is already utilized in coronary stents to prevent the recurrence of thrombosis and restenosis [20]. Our findings, which demonstrate the activation of the mTOR pathway in leg ulcer samples from aPL-positive patients, suggest that this complex may contribute to the development of leg ulcers associated with APS. Given the activation of the mTORC1 pathway in our study group, the potential use of mTORC1 inhibitors in aPL-positive patients with leg ulcers warrants consideration, as these inhibitors could positively influence wound healing by targeting the underlying vasculopathy. The results of this study suggest that the inhibition of mTORC1 in APS-associated leg ulcers may be a potential treatment option for vasculopathy.

For this reason, it might be suggested that vitamin K antagonist treatment be supplemented with an mTOR inhibitor, which would block vasculopathy and reduce the recurrence of APS leg ulcers or further complications. A trial with a topical mTOR inhibitor for ulceration would be worthwhile to provide clinical support for this.

## 4. Materials and Methods

### 4.1. Study Population

In this cross-sectional study, we enrolled patients aged between 18 and 80 years, with a history of lower limb deep vein thrombosis and chronic leg ulcers, and classified them into one of three groups: (1) persistently aPL-positive patients without other systemic autoimmune diseases (primary APS); (2) persistently aPL-positive patients with other systemic autoimmune diseases (secondary APS); and (3) aPL-negative patients (control group). The study protocol was approved by the Regional Research Ethics Committee of the University of Pécs, with the approval number 9878.-PTE 2024. Written informed consent was obtained from all subjects, and the experiments were carried out adhering to Helsinki guidelines. aPL positivity was defined according to the recommendations of the International Society on Thrombosis and Haemostasis as persistent positivity for LAC, aCL IgG/IgM, and/or aβ2GPI IgG/IgM antibodies, with assessments performed at least 12 weeks apart. aPL negativity was established based on negative results for LAC, aCL IgG/IgM/IgA, and aβ2GPI IgG/IgM/IgA antibodies. SLE diagnosis was based on the classification criteria set forth by the American College of Rheumatology. The inclusion criteria for the study were as follows: a history of deep vein thrombosis in the a lower extremity; the presence of chronic, non-healing ulcers on the lower limb of the same side, for which punch biopsies were taken from the edge of the ulcers for histological examination as part of routine diagnostic procedures, in accordance with the recommendation of the European Wound Management Association’s guidelines for leg ulcers and atypical wounds. The exclusion criteria were as follows: an ankle-brachial index below 0.8; histologically confirmed cutaneous vasculitis and/or the biopsy site being affected by another skin disease or infection; the occurrence of acute thrombosis within 30 days prior to the study; immunosuppressive therapy (except hydroxychloroquine, mycophenolate mofetil, azathioprine, or methotrexate) within 6 months prior to the study; the use of prednisolone exceeding 10 mg daily, or an equivalent dose of another corticosteroid, within 30 days prior to the study; previous treatment with mTOR inhibitors (e.g., rapamycin); or confirmed malignancy within 1 year prior to screening (except non-metastatic squamous cell carcinoma or basal cell carcinoma). In the control group, the inclusion criteria necessitated negative results for aPL, APS, and autoimmune diseases, along with the presence of active leg ulcers. The exclusion criteria were consistent with those used for the study group. The inclusion process entailed a comprehensive review of prior laboratory tests and medical records, alongside thorough medical history taking. Throughout the study, only the levels of aCL IgG and IgM, aβ2GPI IgG and IgM, and LAC were considered, in accordance with the diagnostic criteria for APS.

### 4.2. Study Procedures

The study proceeded through the following steps: (1) Clinical data (medical history with details of thromboembolic and APS-related events, as well as autoimmune comorbidities) were collected. (2) A comprehensive review of laboratory test results, including aPL and other autoantibodies, was conducted for patients with chronic leg ulcers, in accordance with the APS diagnostic criteria. (3) The selection of histological samples from patients who had undergone a punch biopsy of the ulcer edge and/or surrounding area for diagnostic purposes according to the EWMA (European Wound Management Association) wound care guidelines. (4) Histological and immunohistochemical examination of the samples: The samples were fixed in 6% buffered formalin and embedded in paraffin. Four-micron-thick sections were stained with hematoxylin–eosin for a general overview, or were prepared for immunohistochemical analysis using Phospho-Akt (Ser473) (D9E) XP^®^ Rabbit mAb and Phospho-S6 Ribosomal Protein (Ser240/244) (D68F8) XP^®^ Rabbit mAb monoclonal antibodies (both by Cell Signaling Technology/Danvers, MA, USA). Breast cancer tissue from the Department of Pathology was used as a positive control for the pS6RP antibody, while a positive control model developed by the manufacturer (SignalSlide^®^ Phospho-Akt (Ser473) IHC Controls #8101, Cell Signaling Technology/USA) was utilized for the pAKT D9E antibody, diluted in the manufacturer-recommended SignalStain Antibody Diluent. The pAKT D9E antibody was applied at a dilution of 1:50, while the p-S6 Ribosomal antibody was diluted to 1:2000 before application using Bond Antibody Diluent (also TRIS buffer). Endothelial cells were visualized using 434-R15 ERG (EP111) Rabbit monoclonal antibody (Cell Marque™/Rocklin, CA, USA), which was applied at a dilution of 1:200.

## Figures and Tables

**Table 1:** aPL antibody levels as well as ANA and ENA screen results for primary APS patients (1–5) and secondary APS patients (6–10). SLE: systemic lupus erythematosus; MCTD: mixed connective tissue disease; NDC SLE type: non-differentiated collagenosis SLE type; NDC Sjögren sy type: non-differentiated collagenosis Sjögren syndrome type. LAC: lupus anticoagulant; aCL: anticardiolipin antibody; aβ2GPI: anti–β2 glycoprotein I; ANA: antinuclear antibody; ENA: extractable nuclear antigen.

Patient	Gender	Age	LAC (0.80–1.20)	aCL (U/mL)		aβ2GPI (U/mL)		ANA (U/mL) (<20)	ENA (<1)
				IgG (<10.0)	IgM (<7.0)	IgG (<8.0)	IgM (<8.0)		
1.	male	32	1.55 1.41 2.18 1.98 1.88 1.83	negative	negative	negative	negative	10.4 14.5 9.7 11.0	0.8 0.1
2.	male	39	1.4 1.26 1.30	negative	negative	negative	negative	12.6	0.1
3.	female	59	1.4 1.6 1.5	negative	negative	negative	negative	4.0	0.1
4.	female	72	1.48 1.26	negative	negative	negative	negative	8.0	0.3
5.	female	56	1.25 1.25	negative	negative	negative 2.5	negative <1.0	3.2 3.7	0.3 0.1
6. SLE	female	56	2.09 2.34 2.60 2.95 3.03 2.84 2.66	>100 >100 96.8 83.7 >100 93.6 99.9	3.1 10.9 2.6 3.2 3.4 2.0 2.0	>100 >100 74.0 98.7 >100 82.8 80.9	2.1 2.4 89.0 3.2 3.1 1.9 1.9	>100 >100 >100 >100	0.3 0.1
7. NDC SLE type	female	48	4.01 4.94	62.9 43.2	24.1 31.5	30.6 20.8	27.4 22.0	9.8	1.3
8. NDC Sjögren sy type	female	73	2.2 1.8 2.0 2.67 2.27 2.66 3.19	6.4 5.5 5.0	14.0 11.4 11.8	7.7 6.2 4.7	15.7 20.4 17.5	55.0 22.1	0.5
9. NDC Sjögren sy type	female	56	2.37 2.47 2.24	58.0 58.6	79.9 59.2	62.3 37.2	73.7 54.5	>100	0.3
10. MCTD	female	61	2.20 1.27	negative	negative	negative	negative	176.7 168.5 38.1 39.7 >100	2.8

**Table 2:** Overview of epidermal phospho-S6 and phospho-AKT expression in leg ulcer samples from patients with primary APS (pAPS) and secondary APS (sAPS). Expression (+), no expression (-).

	Epidermal pS6 Expression	Endothelial pS6 Expression	Epidermal pAKT Expression	Endothelial pAKT Expression
pAPS				
1.	+	+	-	-
2.	+	+	-	-
3.	+	+	-	-
4.	+	+	-	-
5.	+	+	-	-
sAPS				
1.	+	+	-	-
2.	+	+	-	-
3.	+	+	-	-
4.	+	+	-	-
5.	+	+	-	-

## Data Availability

All original data are available from the corresponding author upon request.
